# Effect of Curcumin on Attenuation of Liver Cirrhosis via Genes/Proteins and Pathways: A System Pharmacology Study

**DOI:** 10.3390/nu14204344

**Published:** 2022-10-17

**Authors:** Ali Mahmoudi, Stephen L. Atkin, Tannaz Jamialahmadi, Maciej Banach, Amirhossein Sahebkar

**Affiliations:** 1 Student Research Committee, Faculty of Medicine, Mashhad University of Medical Sciences, Mashhad, Iran; 2 Department of Medical Biotechnology and Nanotechnology, Faculty of Medicine, Mashhad University of Medical Sciences, Mashhad, Iran; 3 School of Postgraduate Studies and Research, RCSI Medical University of Bahrain, Busaiteen, Bahrain; 4 Applied Biomedical Research Center, Mashhad University of Medical Sciences, Mashhad, Iran; 5 Surgical Oncology Research Center, Mashhad University of Medical Sciences, Mashhad, Iran; 6 Department of Preventive Cardiology and Lipidology, Medical University of Lodz (MUL), 93-338 Lodz, Poland; 7 Cardiovascular Research Center, University of Zielona Gora, 65-417 Zielona Gora, Poland; 8 Biotechnology Research Center, Pharmaceutical Technology Institute, Mashhad University of Medical Sciences, Mashhad, Iran; 9 Department of Biotechnology, School of Pharmacy, Mashhad University of Medical Sciences, Mashhad, Iran

**Keywords:** liver cirrhosis, curcumin, protein–protein interaction, biological process, gene expression

## Abstract

Background: Liver cirrhosis is a life-threatening seqsuel of many chronic liver disorders of varying etiologies. In this study, we investigated protein targets of curcumin in liver cirrhosis based on a bioinformatics approach. Methods: Gene/protein associations with curcumin and liver cirrhosis were probed in drug–gene and gene–diseases databases including STITCH/DGIdb/DisGeNET/OMIM/DISEASES/CTD/Pharos and SwissTargetPrediction. Critical clustering groups (MCODE), hub candidates and critical hub genes in liver cirrhosis were identified, and connections between curcumin and liver cirrhosis-related genes were analyzed via Venn diagram. Interaction of hub genes with curcumin by molecular docking using PyRx-virtual screening tools was performed. Results: MCODE analysis indicated three MCODEs; the cluster (MCODE 1) comprised 79 nodes and 881 edges (score: 22.59). Curcumin database interactions recognized 318 protein targets. Liver cirrhosis genes and curcumin protein targets analysis demonstrated 96 shared proteins, suggesting that curcumin may influence 20 candidate and 13 hub genes, covering 81% of liver cirrhosis critical genes and proteins. Thirteen shared proteins affected oxidative stress regulation, RNA, telomerase activity, cell proliferation, and cell death. Molecular docking analysis showed the affinity of curcumin binding hub genes (Binding affinity: ΔG < −4.9 kcal/mol). Conclusions: Curcumin impacted on several critical liver cirrhosis genes mainly involved in extracellular matrix communication, focal adhesion, and the response to oxidative stress.

## 1. Introduction

Liver cirrhosis is the terminal step of many chronic liver disorders with varied etiologies such as fatty liver disease and hepatitis [1]. It has long been recognized as a considerable risk factor for hepatocellular cancer and a leading cause of liver disease-related morbidity and death [2]. Cirrhosis has increased by 1.5-fold to two-fold in the last two decades, and chronic liver disease and cirrhosis are estimated to be responsible for two million deaths globally each year [3]. The prognosis for decompensated liver cirrhosis is poor, with an overall survival rate of 2–4 years, lower than several oncological illnesses [4]. In liver cirrhosis/fibrosis, hepatic stellate cells (HSCs) are critical in initiating and in the development of this late-stage complication, with the activation and transdifferentiation of HSCs into myofibroblasts in response to liver damage, resulting in the generation of abundant extracellular matrix (ECM) proteins such as smooth muscle actin (SMA) [5,6]. A wide range of genes/proteins and biological processes and pathways are involved in this process and the progression to liver cirrhosis [7,8,9,10,11]. The progress of cirrhosis commonly occurs after a protracted period of inflammation. In this process, the healthy liver parenchyma is replaced with fibrotic tissue and renewing nodules, which causes portal hypertension. The condition progresses from an asymptomatic level (compensated cirrhosis) to a symptomatic level (decompensated cirrhosis), leading to hospitalization, reduced quality of life, and a high death rate. Disease outcomes are influenced by systemic inflammation, liver failure, and progressive portal hypertension. The treatment of the causes and effects of liver cirrhosis are the main focus of care, while liver transplantation may be necessary [12]. However, different genes and pathways are related to inflammation and fibrosis such as tumor necrosis factor (TNF), interleukin 17 A (IL-17 A), interleukin 1 beta (IL-1β), interleukin 6 (IL-6), transforming growth factor beta (TGF-β), phosphatidylinositol 3-kinase (PI3K)/protein kinase B (AKT) signaling pathway, and toll-like receptor 4 (TLR4) mediated activation of the nuclear transcription factor κB (NF-κB) signaling pathway that are the targets of curcumin. In this case, a bioinformatics study was designed to determine curcumin’s possible effect on liver cirrhosis [13,14,15,16].

Since ancient times, turmeric has been proposed as a natural cure for a variety of diseases. Curcumin, a natural chemical extracted from the rhizome of *Curcuma longa* (turmeric), has been demonstrated to have antioxidant, antiproliferative, anti-inflammatory, anticancer, cardioprotective, analgesic, antidiabetic, and hepatoprotective properties [17,18,19,20,21,22,23,24,25,26,27,28,29,30,31,32,33]. Numerous in vitro, in vivo, and clinical studies have reported that curcumin may have a beneficial effect on liver cirrhosis [2,34,35,36,37,38,39]. For example, two randomized clinical trials indicated that curcumin supplementation reduced disease activity ratings and cirrhosis severity in individuals with cirrhosis. According to the chronic liver disease questionnaire (CLDQ), The Liver Disease Symptom Index (LDSI), and the Health Survey (SF-36) categories, curcumin enhances quality of life (QoL) in liver cirrhotic patients [2,39]. Curcumin is a pleiotropic compound that may directly or indirectly influence multiple genes/proteins and related pathways involved in inflammatory cytokines, oxidative stress response, growth factors and their receptors, enzymes, adhesion molecules, and apoptosis-related cell cycle proteins; therefore, curcumin may ameliorate liver cirrhosis through a number of mechanisms that are not fully defined [40,41].

Virtual screening based on systems biology methodology has recently garnered interest in target deconvolution studies and the exploration of therapeutic targets in various diseases [41,42,43,44].

**Figure 1:**
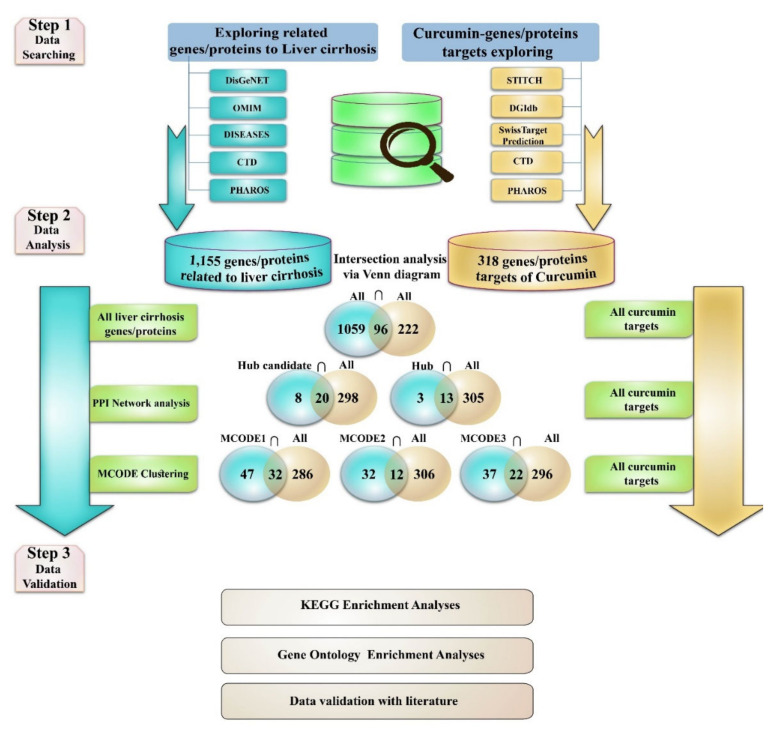
A summary of the study process undertaken in the current study. This research was planned in three main sections including exploring the gene/protein target for curcumin and liver cirrhosis, analyzing the two obtained datasets, and investigating the biological process and pathways related to identified important intersection protein/genes (PPI = protein–protein interaction).

In this study, we hypothesized that the positive effect of curcumin on the end-late injury of the liver in various diseases (liver fibrosis, cirrhosis) reported in previous investigations was through curcumin exerting its effect through multiple genes/proteins and biological pathways. Therefore, we investigated the possible protein targets of curcumin in liver cirrhosis based on a bioinformatics approach. An overview of our research process is shown in Figure 1.

## 2. Methods

### 2.1. Exploring Liver Cirrhosis-Protein/Gene Associations

We used open-access databases including DisGeNET including Online Mendelian Inheritance in Man (OMIM), DISEASES, Comparative Toxicogenomics Dataset (CTD), and Pharos to investigate proteins associated with liver cirrhosis. OMIM (http://omim.org; accessed on 24 April 2022) is a thorough and comprehensive database including information about human phenotypes and genes as well as their associations [45]. DisGeNET (https://www.disgenet.org; accessed on 24 April 2022) is a database containing a list of genes linked to various illnesses, detailing their associations. CTD (http://ctdbase.org; accessed on 24 April 2022) is a manually maintained database that contains information on gene–chemical interactions and their links to disorders [46]. DISEASES (https://diseases.jensenlab.org; accessed on 24 April 2022) is a weekly updated database that contains diseases and gene relationships from computer text mining and manually selected literature [47]. Pharos is an intermediate web source of the Knowledge Management Center (KMC) for assaying the drug target genes and ligands, and it also retains valuable information about the relation of the genes with numerous diseases [48]. We obtained and combined all of the proteins/genes connected to liver cirrhosis from these sources.

### 2.2. Protein–Protein Interaction (PPI) Network and MCODE Analysis of Proteins/Genes Related to Liver Cirrhosis

**Figure 2:**
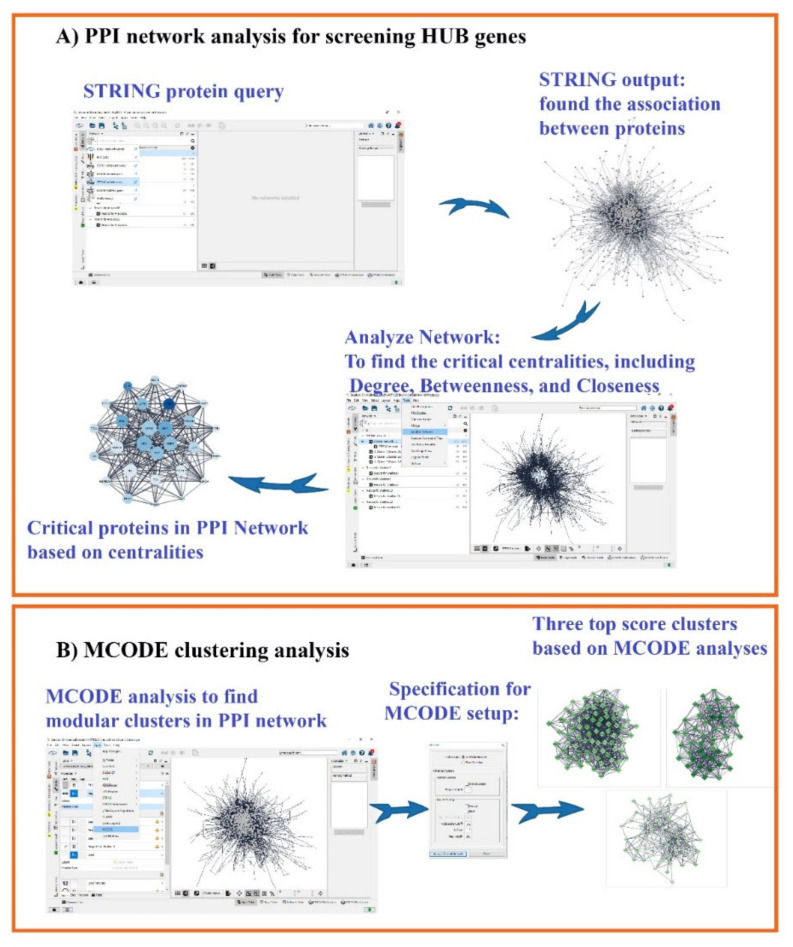
The methodology for screening hub genes through analyzing centralities (**A**) and modular clusters via MCODE analysis (**B**). PPI—protein–protein interactions.

We created a PPI network with liver cirrhosis-related genes obtained from the databases noted above to find the most critical genes (hub genes) and Molecular Complex Detection (MCODE) related to liver cirrhosis. PPI networks were constructed with Cytoscape software (version 3.9.1) and stringApp (version 1.7.0) plugin. A high confidence score >0.7 and the limitation of species to “*Homo sapiens*” were considered. STRING is a web source containing validated and predicted protein associations comprising physical and functional protein–protein interactions [49]. To achieve the most critical hub genes, we used the NetworkAnalyzer version 4.4.8 plugin on Cytoscape software to survey hub gene candidates and hub genes. The hub candidate was determined by exerting 20 percent filtering on the three critical centralities (DegreeCentrality, ClosenessCentrality, and BetweennessCentrality), and the hub genes were determined based on the highest score in these centralities. We selected the 20 highest proteins in each centrality and then assessed the intersection between them to obtain the hub genes. The Degree and Closeness/Betweenness are factors of the topological algorithms and shortest paths, respectively. The MCODE (version 2.0.0) plugin for Cytoscape was utilized to cluster the gene/protein in the PPI network (degree cut-off = 4, haircut off, k-core = 2, node score cut-off = 0.2, and maximum depth = 100). Subsequently, we displayed the specificity of hub candidates related to liver cirrhosis on Cytoscape software using the stringAPP plugin. An illustration of analyzing the PPI network to obtain hub genes and modular clusters in the PPI network by Cytoscape software is depicted in Figure 2A,B.

### 2.3. Curcumin and Target Search

We probed the molecular targets of curcumin in five popular databases (STITCH, DGIdb, SwissTargetPrediction, Pharos, CTD). The STITCH database (http://stitch.embl.de; accessed on 29 April 2022) is a tool for determining chemical–protein interactions including over 9,640,000 proteins from 2031 species [50]. A high confidence (0.700) cut-off and the limitation of the species to Homo sapiens were used. Drug–gene interaction databases (DGIdb) are web-based storage databases that include critical information about drug–gene/protein interactions acquired from a variety of sources including PharmGKB, Therapeutic Target Database (TTD), DrugBank, Drug Target Commons Chembl, and others [51]. SwissTargetPrediction is a web service that uses a mix of 2D and 3D similarity metrics with known ligands to predict the targets of bioactive compounds [52]. We selected all gene/protein targets for curcumin in those databases.

### 2.4. Estimation of Curcumin Targets with the Genes/Proteins Connected with LIVER Cirrhosis

To exclude spelling errors in gene symbols, we first converted all of the genes/proteins obtained from all databases to the UniProt Accession via https://biodbnet-abcc.ncifcrf.gov/db/db2db.php; accessed on 25 April 2022, then applied the Venn diagram.

We evaluated the intersection of the datasets (gene association with liver cirrhosis and curcumin targets) in three phases to find important protein targets of curcumin in liver cirrhosis disease:

1 Evaluation of the curcumin targets with all achieved proteins/genes associated with liver cirrhosis (All liver cirrhosis connection genes ∩ curcumin targets).
2 Evaluation of the curcumin targets with important proteins/genes obtained based on PPI network analysis (hub/candidate hub genes connection liver cirrhosis ∩ curcumin targets).
3 Evaluation of the curcumin targets with important proteins/genes obtained based on MCODE analysis (MCODEs connection with liver cirrhosis ∩ curcumin targets). The intersection across these lists was conducted with online Venn diagram software (https://bioinfogp.cnb.csic.es/tools/venny; accessed on 24 April 2022).

We also investigated the possible direct interaction of hub genes with curcumin by molecular docking. We first downloaded the 3D structure of the hub genes from the RCSB PDB web server. The 2D structure of curcumin (SDF) was determined from the PubChem source. For docking operations, we used PyRx-virtual screening tools. Before the docking, we first prepared the structure of macromolecules using UCSF Chimera software (version 1.8.1) and then we performed docking of the macromolecules and curcumin compound in PyRx software. The Protein Data Bank (PDB) is the first open-access digital data resource with information about the 3D structure of biological molecules in all organisms [53]. PubChem is an open web server at the National Institutes of Health (NIH), which is a resource with information about the chemical structures [54]. PyRx software is used for Computational Drug Discovery to probe compounds against potential biological targets [55]. UCSF Chimera software is used to analyze the structures of the molecules [56]. The results of molecular docking analysis are reflected in the binding energy (ΔGbind). Energy binding less than −5 kJ·mol^−1^ (−1.19503 kcal/mol) refers to the certain binding activity of the ligand and the targets [57].

### 2.5. Gene Ontology and KEGG Pathways Enrichment Analysis

Functional enrichment analysis (GO) and biological pathway (KEGG) were accomplished using the freely available ClueGO plugin for Cytoscape software, and the charts are presented with Excel 2013 software. ClueGO was used to interpret the interrelations of the gene/protein sets and functional groups in biological networks [54]. GO is defined in three stages: Biological Process, Cellular Component, and Molecular Function [58]. Likewise, KEGG is a repository database that interprets genomic, diseases, pathways, chemical, and systemic functional data [59].

## 3. Results

### 3.1. Gene Related to Liver Cirrhosis and PPI Network Analysis 

**Figure 3:**
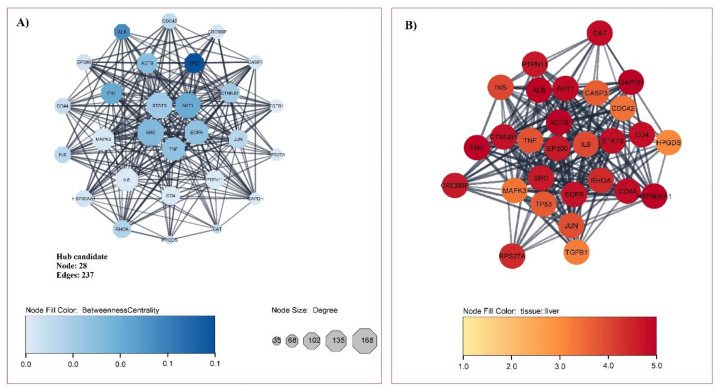
(**A**) Hub protein/gene candidate interaction network constructed by filtering proteins/genes by high-level centralities using Cytoscape software. Twenty-eight candidate hubs with 237 edges were identified. The Degree is depicted with the size of nodes, and Betweenness is depicted with the intensity of the node color. (**B**) The amount of presentation of proteins/genes related to liver cirrhosis liver tissue. The intensity of the color indicates the amount of presentation in the liver.

We found 1155 associated genes with liver cirrhosis by probing gene/protein-disease association databases. The PPI network constructed with the stringApp plugin possesses 906 nodes and 9647 edges with PPI enrichment *p*-value: <1.0 × 10^−16^. The subsequent network analysis of centralities (DegreeCentrality, ClosenessCentrality, and BetweennessCentrality) on Cytoscape distinguished 28 candidate hub genes and 16 hub genes. The hub genes included tumor protein 53 (TP53), albumin (ALB), fibronectin 1 (FN1), serine/threonine kinase 1 (AKT1), non-receptor tyrosine kinase (SRC), actin beta (ACTB), tumor necrosis factor (TNF), epidermal growth factor receptor (EGFR), signal transducer and activator of transcription 3 (STAT3), catenin beta 1 (CTNNB1), ras homolog family member A (RHOA), jun proto-oncogene (JUN), insulin (INS), CD44, E1A binding protein p300 (EP300), and heat shock protein 90AA1 (HSP90AA1). Candidate hub genes and hub genes are illustrated in Figure 3A and described in Table 1, respectively. The presentation of proteins/genes associated with liver cirrhosis is shown by color intensity in Figure 3B (score 1 to 5) (Figure 3); all of the genes/proteins described had a score of three or higher, and 19 proteins scored more than 4. The scoring of tissue specificity was achieved from TISSUES 2.0 databases as part of the STRING app in Cytoscape software from a score of 1 to 5. The scoring was based on integrating multiple sources of evidence: transcriptomics as well as manually curated and mined from the scientific literature [60].

**Figure 4:**
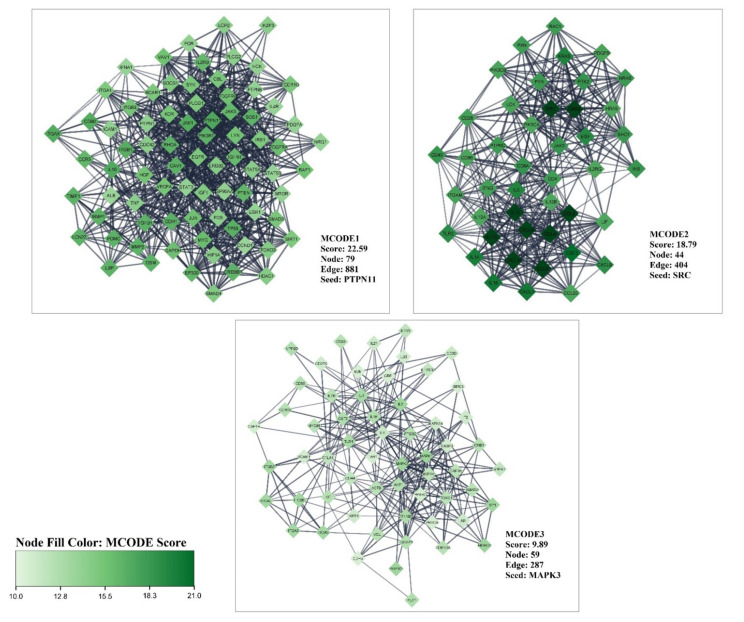
The three top score clusters of genes/proteins related to liver cirrhosis based on MCODE analyses.

Furthermore, in MCODEs analysis, the three highest MCODEs were determined (Figure 4). MCODE 1 had the highest score (22.59) with 79 nodes and 881 edges. MCODE 2, with a score of 18.79, 44 nodes, and 404edges was the second most crucial modular cluster in the PPI network related to liver cirrhosis. PTPN11 and SRC are the seed of MCODE 1 and 2, respectively. The third score cluster comprises 59 nodes and 287 edges with 9.89 score and seed of MAPK3.

### 3.2. Obtained Curcumin Targets and Exploring the Impact of Curcumin on Proteins/Genes Related to Liver Cirrhosis

Screening curcumin targets in the STITCH, DGIdb, SwissTargetPrediction, Pharos, and CTD databases identified 318 capable protein/gene targets.

**Figure 5:**
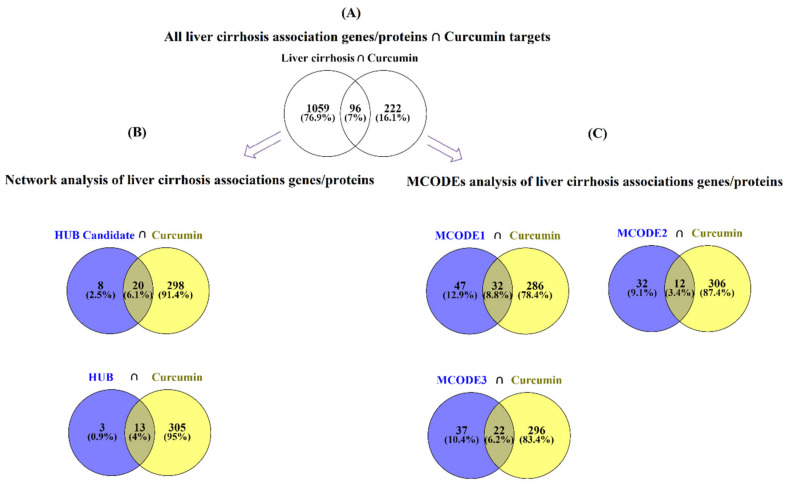
Intersection analysis of the curcumin targets and liver cirrhosis-related genes/proteins using a Venn diagram with (**A**) curcumin targets and all liver cirrhosis association genes, (**B**) curcumin targets and hub candidate/hub genes, (**C**) curcumin targets and MCODEs analysis of liver cirrhosis association genes/proteins.

Intersection analysis with a Venn diagram of all proteins/genes associated with liver cirrhosis and the protein targets of curcumin exhibited 96 shared proteins (Figure 5A).

The Venn diagram for hub candidates and hub proteins/genes associated with liver cirrhosis demonstrated that 20 and 13 proteins/genes, respectively, connected with curcumin (Figure 5B). The 13 hub genes that curcumin could interact with included AKT1, ALB, CTNNB1, EGFR, EP300, FN1, HSP90AA1, INS, JUN, SRC, STAT3, TNF, and TP53. This result indicated that although curcumin covered only 8.31% of all liver cirrhosis protein targets, it could cover 81.25% of the most critical proteins/genes based on PPI network analysis.

Comparing the curcumin targets with the three top-scored MCODE clustering of liver cirrhosis-related proteins/genes revealed that curcumin targeted 32 crucial proteins/genes of MCODE1, the highest covering of proteins/genes among other MCODEs. Interestingly, MCODE3 and 2 targeted 22 and 12 proteins/genes, respectively, suggesting that curcumin targets critical proteins/genes that belong to the most important clustered proteins/genes associated with liver cirrhosis (Figure 5C).

**Figure 6:**
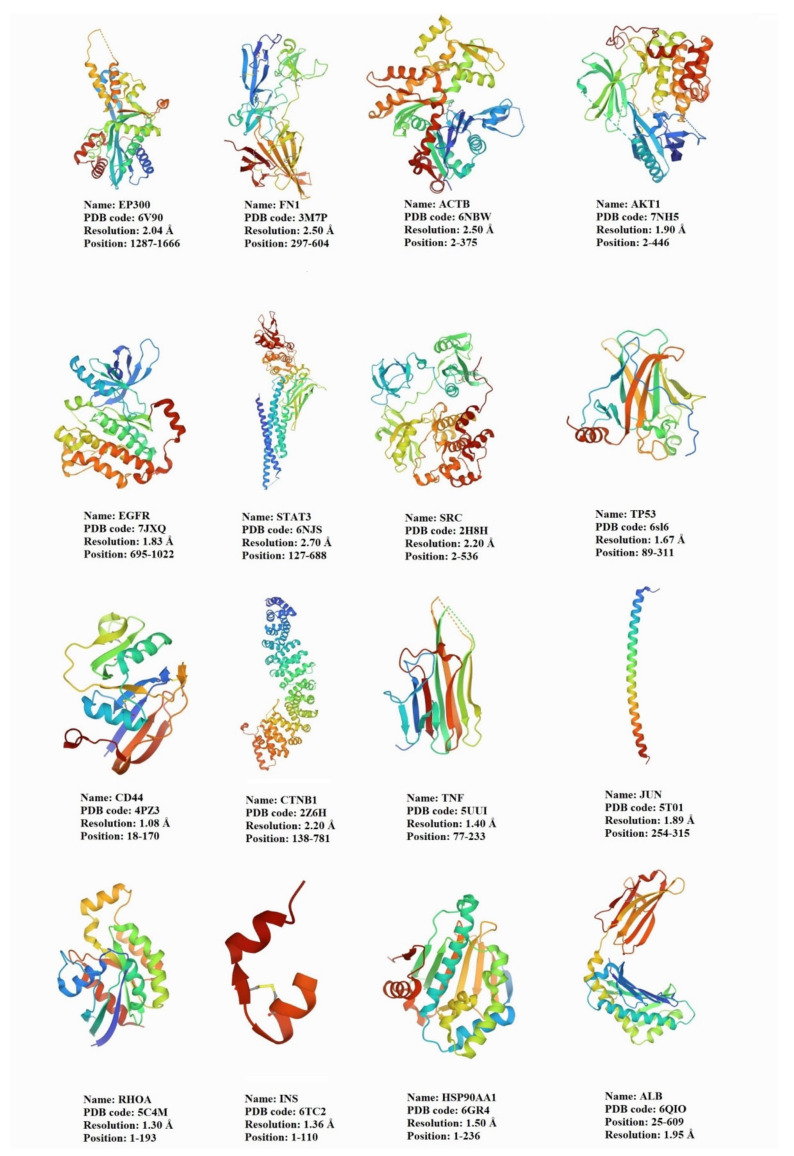
The structure of proteins encoded by 16 hub genes obtained from the RCSB PDB databases.

**Figure 7:**
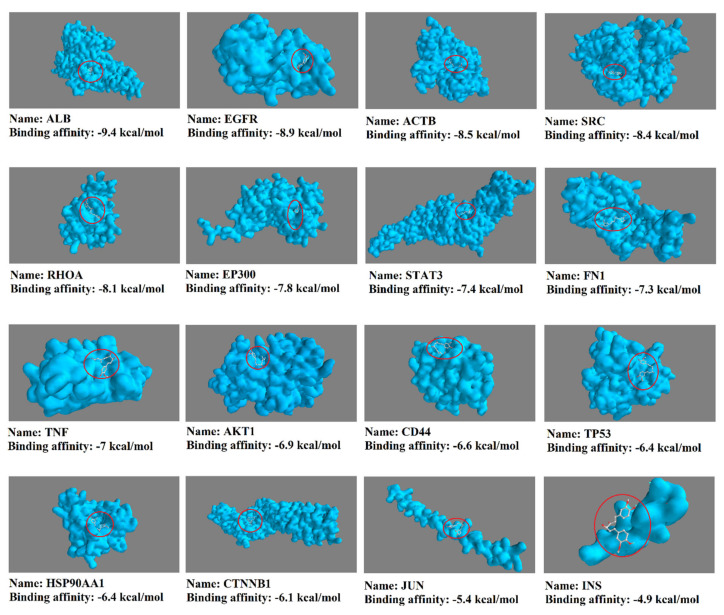
The possible interaction of curcumin with proteins encoded by 16 hub genes using the molecular docking method.

To analyze the molecular docking of curcumin against 16 hub genes/protein targets, we first identified the 3D structure of the targets (Figure 6). Then, in UCSF Chimera, other chains, ligands, non-complexed ions, and solvent were removed. The structure of curcumin was obtained in PubChem with CID: 969516. As shown in Figure 7, the docking result indicated that curcumin is a pleiotropic and flexible compound that could interact with most of the targets with a binding affinity within the minimum Δ*G* energy.

### 3.3. GO and KEGG Enrichment Analyses of Shared Proteins

**Figure 8:**
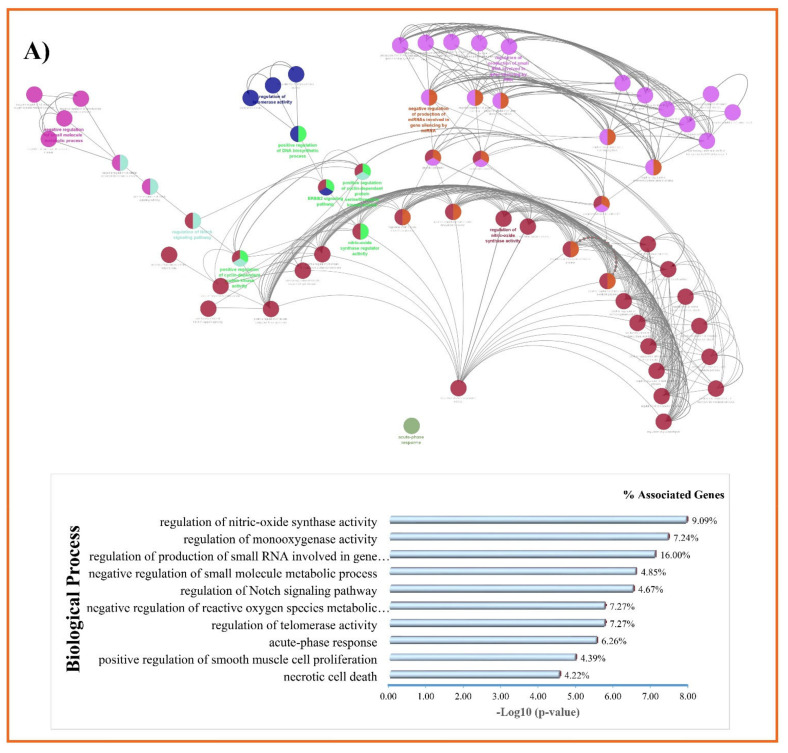

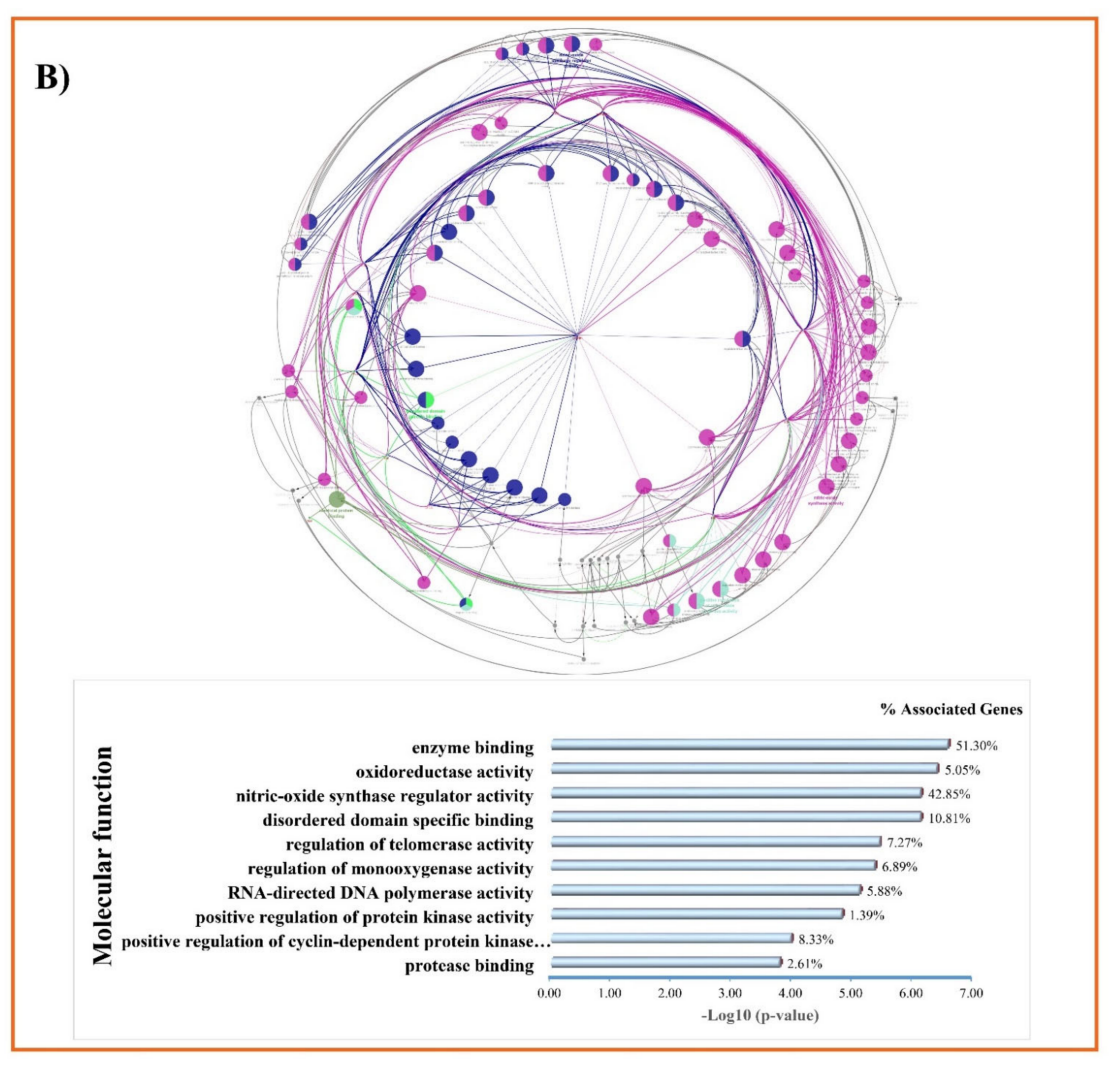

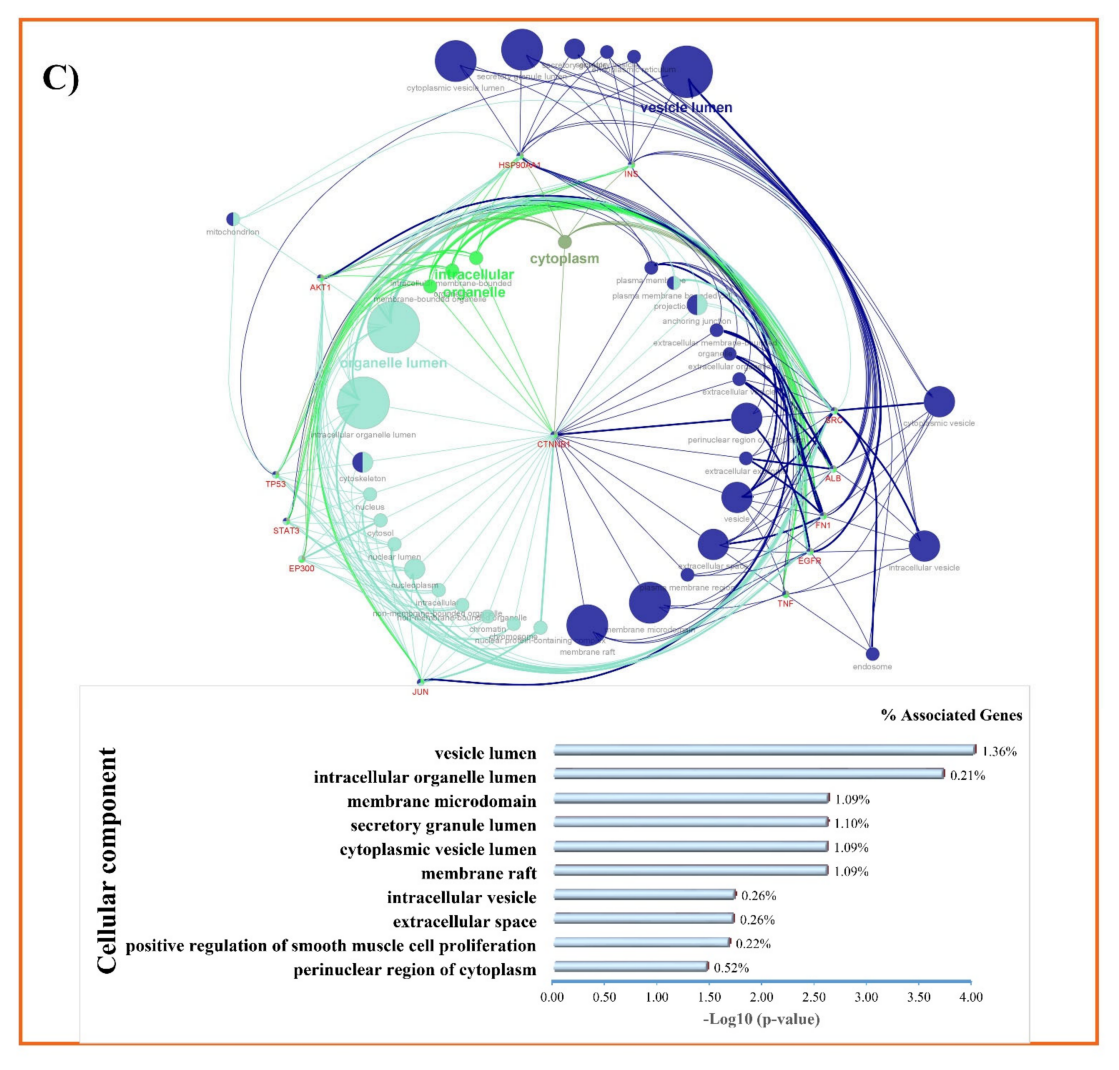

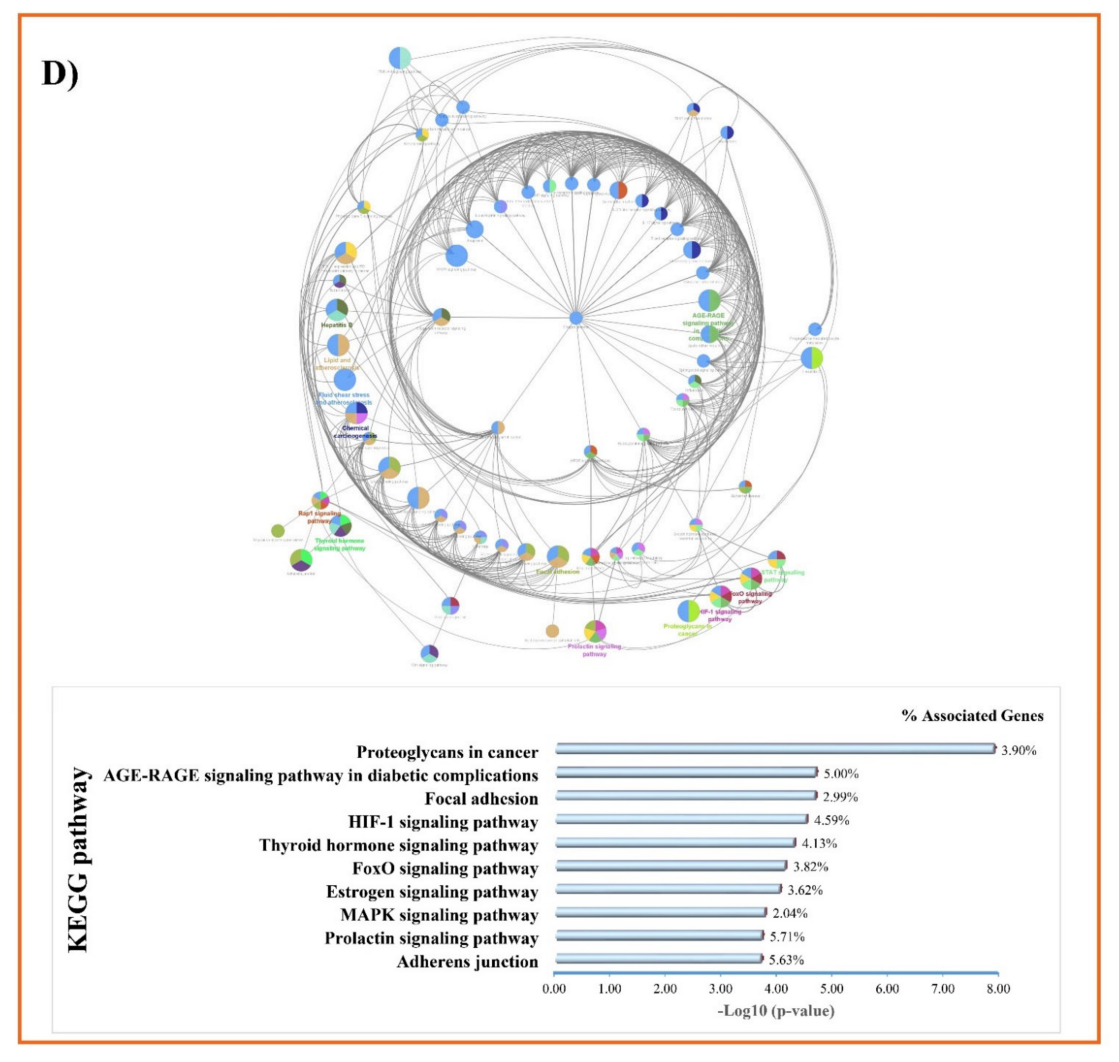
Gene Ontology enrichment analysis of the 13 shared protein targets (hub associated with liver cirrhosis ∩ curcumin targets) using ClueGO. (**A**) The ten highest adjusted *p*-value biological process; (**B**) the ten highest adjusted *p*-value molecular function; (**C**) the ten highest adjusted *p*-value cellular compound; (**D**) the ten highest adjusted *p*-value biological pathway (KEGG pathway) enrichment. Associated genes % indicates the percentage of gene sets present in each functional term. For example, if 50 genes are involved in a pathway, and ten genes of our gene set are involved in this pathway, the associated genes % equals 5%.

Functional enrichment analysis of 13 shared proteins (intersection of hub genes associated with liver cirrhosis ∩ curcumin targets) at the biological process level suggested the regulation of nitric-oxide synthase activity with 9.09% of associated genes percent and *p*-value of 1.26 × 10^−8^, the regulation of monooxygenase activity with 7.24% of associated genes percent and *p*-value of 3.97 × 10^−8^, and the regulation of the production of small RNA involved in gene silencing by RNA 4.04% of associated genes percent and *p*-value of 8.91 × 10^−8^ (Figure 8A). In addition, the analysis demonstrated that these shared proteins were mainly applied in enzyme binding with 5.13% of associated genes percent and *p*-value of 2.78 × 10^−7^; oxidoreductase activity with 5.05% of associated genes percent and *p*-value of 4.23 × 10^−7^; nitric-oxide synthase regulator activity with 42.85% associated genes percent and *p*-value of 7.93 × 10^−7^, and the regulation of telomerase activity with 7.27% of associated genes percent and *p*-value of 3.83 × 10^−6^ (Figure 8B). Moreover, vesicle lumen with 1.36% of associated genes percent and *p*-value: 1.01 × 10^−4^, intracellular organelle lumen with 0.21% of associated genes percent and *p*-value of 2.03 × 10^−4^, membrane microdomain with 1.09% of associated genes percent and *p*-value of 0.0026, and secretory granule lumen with 1.10% of associated genes percent and *p*-value of 0.0026 were detected under cellular components (Figure 8C).

KEGG enrichment revealed that the pathways with the highest adjusted *p*-value were associated with 13 shared proteins including proteoglycans in cancer with 3.90% of associated genes percent and *p*-value of 1.39 × 10^−8^, the AGE-RAGE signaling pathway in diabetic complications with 5.00% of associated genes percent and *p*-value of 2.23 × 10^−5^, the focal adhesion with 2.99% of associated genes percent and *p*-value of 2.29 × 10^−5^, and the HIF-1 signaling pathway with 4.59% of associated genes percent and *p*-value of 3.28 × 10^−5^ (Figure 8D).

## 4. Discussion

Hepatic cirrhosis is a global and increasing health care problem with high morbidity and mortality. There is a need for anti-fibrotic treatments to modulate and prevent disease [61]. In hepatic fibrosis, the level of extracellular matrix constituents such as fibronectin, proteoglycans, and laminin increased in reaction to liver damage. Leukocyte recruitment occurs after the injury and needs multiple adhesion molecules. By generating nitric oxide and inflammatory cytokines, macrophages and monocytes play a role in inflammatory responses. Stellate cells and extracellular matrix-producing cells undergo a process of activation due to chronic tissue injury, distinguished by motility, proliferation, contractility, and extracellular matrix synthesis [62]. Curcumin is a potent compound that significantly impacts numerous genes/proteins in various biological and pathway processes [41] and we show here that several important genes implicated in liver cirrhosis based on topological algorithms and the shortest paths analysis of the PPI network were identified. We also showed that these targets could interact with and are potentially modulated by curcumin. Molecular docking also indicated that curcumin has a high affinity in interaction with hub genes. The biological processes analysis of the acquired targets is mainly concerned with oxidative stress regulation, small RNA production, telomerase activity, cell proliferation, and necrotic cell death. In our study, various important pathways were highlighted based on enrichment analysis, and some of them have been widely studied for liver cirrhosis [63,64,65,66].

In this study, the proteoglycans in the cancer pathway were significantly identified with a set of genes that included AKT1, CTNNB1, EGFR, FN1, SRC, STAT3, TNF, and TP53, which was the highest pathway in biological pathways analysis that was influenced by curcumin in liver cirrhosis. Proteoglycans are macromolecules made up of a protein core with sugar chains linked covalently. They can be found on the cell surface and in the extracellular matrix (ECM) of living organisms. Syndecan-1 is the most common transmembrane proteoglycan in normal liver [63]. During the fibrogenesis process, proteoglycans may play a role in controlling cellular processes and preserving the supramolecular architecture of the extracellular matrix in both normal and diseased livers [67]. Furthermore, it documented that proteoglycans increase in liver cirrhosis [63]. Transforming growth factor β1 (TGF-β1) is a potent pro-fibrotic cytokine [68] that in our analysis was found to be a hub candidate that might be influenced by curcumin. Decorin is a short leucine-rich proteoglycan that binds to TGF-1 with high affinity and blocks it from interacting with pro-fibrotic receptors. Decorin has previously been demonstrated to have a protective function in liver fibrogenesis since the genetic ablation of decorin in mice results in increased poor matrix degradation, matrix deposition, and the activation of hepatic stellate cells, the major indices of fibrotic tissue [61]. Furthermore, proteoglycans such as endocan and syndecan-1 in the serum of patients with alcoholic cirrhosis are potential prognostic biomarkers for liver cancer [69]. The previous research by Kovalszky et al. in 1998 on animal models with liver fibrosis indicated that proteoglycans participate in fibrogenesis. Hepatocytes synthesize the bulk of liver-specific heparan sulfate proteoglycans such as fibroglycan and syndecan-1. Perlecan and decorin are extracellular matrix proteoglycans produced by non-parenchymal liver cells. In a healthy liver, the amount of proteoglycans is relatively modest, but it rises rapidly in liver fibrosis [70]. On the other hand, in vitro studies indicate that curcumin could inhibit the apoptosis of chondrocytes, repress the induction of proteoglycans and metal metalloproteases, cyclooxygenase expression, prostaglandin E-2, and inflammatory factors in chondrocytes [71]. In mesenchymal stem cell progenitors, curcumin markedly increases cartilage-specific proteoglycans (CSPGs), collagen type II production, activates MAPKinase signaling, β1-integrin, and represses cyclooxygenase-2 and caspase-3. It is suggested that curcumin may assist in establishing a microenvironment [72].

The AGE-RAGE signaling pathway in the diabetic complications signaling pathway was significantly found in an enrichment analysis with the AKT1, FN1, JUN, STAT3, and TNF genes indicated as hub genes influenced by curcumin. These results indicate that curcumin is involved in the pathway associated with liver cirrhosis. The activation of advanced glycation end products (AGEs) through their receptors (RAGE) causes an increase in reactive oxygen species (ROS), which is hypothesized to contribute to hepatic fibrosis through hyperglycemia [73]. Hepatic fibrosis was linked to the activation of the AGE receptor (RAGE) in hepatic stellate cells. The RAGE signaling system is activated by NADPH oxidase, which causes a rise in reactive oxygen species (ROS), matrix metalloproteinase-9 (MMP-9), alpha-smooth muscle actin (alpha-SMA), and RAGE [64]. The liver is one of the principal organs engaged in inactivating AGEs and this function is impaired in individuals with hepatic cirrhosis [74]. Furthermore, a previous study on patients with hepatitis c-related fibrosis supported that AGEs stimulate autophagy and activation of HSCs that are involved with fibrosis [75]. Curcumin has been shown to suppress fibrosis by hindering the RAGE-mediated effects of AGEs such as raised oxidative stress, inflammation, and cell proliferation in hepatic stellate cells via PPAR-γ activation [76]. PPAR- is a central transcriptional regulator that inhibits multiple transcription factors linked to α-SMA production and hepatic fibrosis including activated protein-1 (AP-1) and specialized protein-1, and nuclear factor-kappa B (NF-κB) [77]. Curcumin also inhibits the effects of AGEs in hepatic stellate cells by disrupting leptin signaling and triggering the transcription factor NF-E2 p45-related factor 2 (Nrf2), which resulted in an increase in cellular glutathione and a decrease in oxidative stress [78]. A recent transcriptomic analysis reported that curcumin suppresses the extracellular matrix (ECM)–receptor interaction and AGE-RAGE signaling pathway in the diabetic retina [79]. Furthermore, a recent bioinformatic analysis proposed that curcumin could ameliorate fatty liver diseases by impacting the AGE-RAGE signaling pathway [41].

The focal adhesion pathway encompasses several genes, and KEGG analysis demonstrated that AKT1, CTNNB1, EGFR, FN1, JUN, and SRC were strongly related to curcumin compounds that might be influenced by liver cirrhosis in our study.

Cell–matrix adhesions are critical in a variety of biological activities including cell motility, differentiation, proliferation, cell survival, and gene modulation. Focal adhesions arise at the cell-extracellular matrix contact sites, where bundles of actin filaments are tethered to transmembrane receptors of the integrin family via a multi-molecular complex of protein junctions. Focal adhesion includes signaling molecules such as phosphatases and protein kinases or structural links between the actin cytoskeleton and membrane receptors [80,81,82]. Focal-adhesion-kinase (FAK) is one of the most significant targets for hepatic stellate cells and liver fibrosis development in vivo. In fibrotic living tissues, FAK activation is linked to enhanced expression of α-SMA and collagen. TGF-β1 induces FAK activation in a dose and time-dependent manner. Inhibiting FAK activation prevents collagen and α-SMA expression and decreases the production of stress fibers, which reduces liver fibrosis [65]. An animal study in 2020 reported that animals lacking FAK in liver epithelial cells compared to the controls had more severe liver damage and fibrosis. They also showed that the activation of numerous pro-fibrotic pathways including the hedgehog/smoothened pathway was associated with FAK-deficient animals and promoted matrix stiffness independently [83]. A proteomic and transcriptomic study reported that focal adhesion and actin cytoskeleton pathways were significantly involved in rats with liver fibrosis [66]. Through the focal adhesion kinase pathway, curcumin may control cellular processes such as migration and proliferation. Curcumin could modulate this pathway by alternating phosphorylation kinases such as MAPK12, AXL, FRK, and TNK2 as well as phosphatases such as INPPL1, PTPRK, and PTPN6 [84]. Others have reported that curcumin treatment hinders the expression of integrin, Nm23, E-cadherin, TIMP-2, metalloproteinase activity, adhesion, and focal adhesion kinase [85], and previous studies have shown that curcumin could specifically downregulate MMP-2, integrin receptors, and FAK activity [86].

Hypoxia-inducible factor 1 (HIF-1) is a heterodimer (Hif-1β and Hif-1α) and is considered to be a pivotal regulation factor that activates several hypoxia-responsive genes and is responsible for cell survival in hypoxia [87]. Previous investigations focused on the role of HIF-1α in the pathogenesis of tumors; however, several reports proposed its role in liver fibrosis by the modulation of a set of genes involved in collagen synthesis and angiogenesis [88,89,90,91]. The HIF-1 signaling pathway was significantly found in enrichment analysis with the AKT1, EGFR, EP300, INS, and STAT3 genes, the hub genes that were also identified as a target of curcumin. The analysis of liver fibrosis in rats induced by carbon tetrachloride (CCl4) indicated that the HIF-1 signaling pathway in a KEGG analysis showed that HIF-1 is significantly involved in liver fibrosis [66]. Another study reported that the development mechanism of liver fibrosis is implicated in the upregulation of HIF-1α transcriptional activity and its interconnected factors, TGF-β and NF-κB, in bile duct ligation-stimulated hepatocytes fibrosis in rats [92].

HIF-1 activates HSCs in a hypoxic environment caused by liver fibrosis and inflammation [93]. It was recently discovered that HIF-1 is controlled by a variety of signaling mechanisms. HIF-1 transcription and synthesis are regulated by the phosphoinositol 3-kinase (PI3K)/Akt pathway, whereas HIF-1 transcription is induced by the mitogen-activated protein kinase (MAPK) pathway [91]. In our study, these two pathways were also observed as the significant pathways in liver cirrhosis regulated with curcumin. Inhibition of PI3K signaling in HSCs reduces collagen production, ECM deposition, and profibrogenic factor expression [94]. Others report that MAPK inhibition inhibits eNOS phosphorylation during hypoxia, resulting in decreased HIF-1 expression [95]. This pathway is also at a crossroads with the focal adhesion pathway, influencing HSC activation and proliferation, which is important for hepatic fibrogenesis [96,97]. Evidence suggests a relation between the HIF-1 and Nrf2. The Nrf2 pathway plays a key role in activating the HIF-1-related activities [98]. Curcumin can affect transcription factors such as NF-ĸB, Nrf2, STAT3, and protein kinases such as Akt and MAPKs [99]; therefore, it might impact the HIF1 signaling pathway. The curcumin regulation of the ubiquitin-proteasomal system (UPS) has been linked to the regulation of cancer-linked apoptotic proteins, inflammatory proteins, cell cycle proteins, transcription factors such as HIF-1, and growth factors related to the inhibition of cancer processes [100]. Curcumin has been demonstrated to downregulate Notch1 expression, hindering hypoxia-induced HIF-1 expression and acting as a possible anticancer drug for treating osteosarcoma [101]. HIF-1 interacts with the intracellular domain of Notch1, which is produced by γ-secretase cleavage and translocates to the nucleus to activate Notch target genes in hypoxia. Furthermore, other research found that curcumin decreased angiogenesis and HIF-1 expression in osteosarcoma cells. Curcumin also inhibits HIF-1 expression by interfering with the p38 MAPK and Akt/mTOR pathways. According to these studies, Akt/mTOR is also a significant upstream regulator of NF-B and GSK3, and as a result, GSK3 and NF-B may play a role in the curcumin inhibition of HIF-1 [102].

Our study also illustrated that curcumin may strongly modulate SRC, which had a strong interaction with low ΔG energy: −8.4 kcal/mol. SCR was recognized as a key protein interaction based on the PPI network analysis with the highest degree score of 168, high Betweenness score of 0.0350, and Closeness score of 0.4612, introducing this gene as one of the vital therapeutic targets for liver cirrhosis. We also observed that SRC was markedly expressed in the liver (score: 4.64). SRC kinase families are non-receptor tyrosine kinases observed in all cell types. However, SRC is demonstrated to be active in renal and pulmonary fibrosis [103,104]. SRC expression varies dramatically depending on the stage of liver cirrhosis [105]. According to Western blot and immune-histochemical analyses, src-related tyrosine kinases were discovered in large numbers in patients with liver cirrhosis and hepatocellular carcinoma, suggesting that SRC-RTK may have a role in the formation and progression of both diseases [106]. Other research suggests that liver cirrhosis might be attenuated by using SRC inhibitors [107]. In cirrhosis patients and thioacetamide (TAA)-induced fibrotic mice livers, SRC expression was shown to be increased, and phospho-SRC was elevated after the activation of hepatic stellate cells. Src inhibition also decreased TGF-β and lowered the expression of α-SMA in primary hepatic stellate cells. The downregulation of smad3 was linked to the anti-fibrotic impact of SRC inhibitors. Furthermore, inhibiting SRC enhanced autophagy flux and protected the liver against fibrosis [103]. An in vitro kinase investigation indicated that curcumin directly inhibits SRC activity [108]. In a study by Yang et al., SRC is an upstream signaling transducer of several genes including JNK and Smad3, and has a close relation with TGFβ1. They also commented that curcumin significantly abolished the TGFβ1-induced CCN2 by hindering the phosphorylations of SRC, Smad3, and JNK, and it also interfered with α-SMA and TGFβ1 expression [109]. Others have reported that curcumin affects the SRC by promoting hypomethylation of the mir-203 promoter and increasing the expression of this microRNA; mir-203 is the target of SRC gene inhibition [110].

AKT is a serine/threonine-protein kinase that has a primary function in the development of liver fibrosis. Akt is composed of three specific isoforms including AKT-1, AKT-2, and AKT-3 [111]. The induction of fibrosis in the liver of mice reported that all AKT forms were triggered with a concomitant rise in the activated forms of mammalian target-of-rapamycin complex 2 (mTORC2) phosphoinositide-dependent kinase-1 (PDK1) and PI3K, leading to the increasing level of inflammatory, fibrogenic, and proliferative genes [112]. An RNA sequencing study on patients with hepatitis B cirrhosis indicated that AKT is associated with liver cirrhosis [58]. The PI3K/Akt pathway plays a key part in apoptosis, motility, and cell growth, and the overexpression and activation of Akt1 and Akt2 boost the K8/18 protein levels [113]. Another investigation demonstrated the abolishing of AKT-mediated phosphorylation of glycogen synthase kinase 3β (GSK3β) on Ser9 in cirrhosis compared to the normal cells [114]. In the cirrhosis liver of rats, phosphorylation of AKT and eNOS were obviously impaired [115]. In several lines of research, curcumin was introduced as a regulator of AKT1. In an in vitro study, the incubation of curcumin in MDA-MB231 cells reduces the level of AKT1 [116]. A recent study showed that when curcumin was combined with bile, it inhibited Akt1 much more effectively [117]. Others reported that the constitutive activation of AKT1 led to the inhibitory effect of curcumin on cell proliferation and migration in human bladder cancer [118]. Using molecular docking, a study to find possible applicable curcumin targets for treating colorectal cancer showed that curcumin could stably combine with EGFR, STAT3, and AKT1. AKT1, the strongest binding to curcumin, was shown in their study [119]. In accordance with our study, EGFR with binding affinity energy: −8.9 kcal/mol, STAT3: −7.4 kcal/mol, and AKT1: −6.9 kcal/mol interacted with curcumin. Here, we showed AKT1 with a Degree of 156, Closeness of 0.4619, and Betweenness of 0.0403, a crucial protein target in liver cirrhosis. Furthermore, it displayed a high specific presentation in the liver tissue (score: 4.88).

Epidermal growth factor receptor (EGFR) is a tyrosine kinase receptor triggered by a variety of ligands, resulting in the activation of a number of signaling pathways that affect differentiation, proliferation, and survival. The EGFR signaling axis has been demonstrated to be important in developing liver diseases such as cirrhosis and liver cancer [120]. Inhibiting EGFR reduces liver fibrosis and HSC activation in NAFLD based on recent research, indicating the dysregulation of EGFR in animal models of liver damage [121,122]. EGFR is phosphorylated in the liver tissues of high-fat diet (HFD) mice with NAFLD. Diet-induced oxidative stress, lipid accumulation, HSC activation, and matrix precipitate were all reduced by inhibiting EGFR. HSC activation was mediated by EGFR, which led to a pro-fibrogenic phenotype [123]. A previous clinical study reported that the positive rates of EGFR expression were 66.22% in liver cancer, 44.00% in liver cirrhosis, and 10.00% in normal liver tissues. The expression of EGFR was remarkably higher in liver cirrhosis than in normal liver tissue [124]. Several molecular dynamics and docking studies showed that curcumin has great potential for anti-EGFR activity [125,126,127,128]. Curcumin exerts its anticancer property by inhibiting proliferation, EGFR phosphorylation, and promoting the degradation of EGFR by ubiquitination and apoptosis [129]. It has also been confirmed that the induction of apoptosis is mediated through the EGFR signaling pathway [130]. A study has shown that curcumin could control EGFR and EGFR downstream signaling molecules containing Akt, STAT3, and ERK1/2 [131]; curcumin inhibited EGFR via efficiently modulating gefitinib-insensitive EGFR degradation [132]. The hydroxyl groups in curcumin and related analogs were important for their biological actions in affecting EGFR expression [133]. In our analysis, EGFR showed great impact in the PPI network-liver cirrhosis with a Degree of 149, Betweenness of 0.0245, and Closeness of 0.4498, which curcumin strongly interacts with and blocks its function with a low binding affinity energy of −8.9 kcal/mol. Furthermore, our acquired data indicates that an EGFR score of 4.75 has high liver specificity.

The signal transducer and activator of transcription-3 (STAT3) perform a critical role in the pathophysiology of liver disorders [4]. STAT3 is a cytoplasmic signal transcription factor that is a member of the Janus kinase (JAK)/STAT pathway and is thought to have an essential role in mediating liver damage [134]. STAT3 activation is an essential signal for liver renewal in cirrhotic livers from the hepatitis C virus (HCV) infection and alcoholics, and suppressing liver renewal in liver cirrhosis might be due to reduced STAT3 activation [135]. Another study reported that in liver cirrhosis, STAT3 DNA-binding is impaired by raising the expression of Pias3 [136]. STAT3 proteins were found in abundance in the nuclei of proliferating biliary epithelial cells and hepatocytes in cirrhosis livers. Although Stat3 proteins are upregulated in cirrhosis, this transcription factor appears to be functionally inactive. Impaired STAT3 activation and restoration may thus contribute to the development of cell damage, which leads to liver cirrhosis [137]. Moreover, others have reported that STX-0119, an inhibitor of STAT3 dimerization, could suppress the progress of liver cirrhosis by obstructing hepatic stellate cell activation [138]. Differing research indicates that curcumin could regulate the STAT3 signaling pathway, with most research focusing on the role of STAT3 in cancers and its inhibition by curcumin. A study reported that curcumin decreased STAT3 phosphorylation and prevented STAT3-mediated signaling [139]. Previously, research in a colitis animal model reported that the phospho-STAT3 activity and DNA-binding activity of STAT3 dimers were significantly decreased following therapy with curcumin [140]. A study showed that expression levels of STAT3 declined after curcumin administration in MDA-MB-231 cells [141]. Research by Hahn et al. in 2018 determined that the cysteine residue 259 of STAT3 is an assumed site for the binding of curcumin. The authors also reported that the α,β-unsaturated carbonyl moiety in curcumin appears critical for binding to STAT3 [142]. Furthermore, several studies have proposed that STAT3 could play a crucial function in inflammation-associated tumorigenesis [143,144]. Curcumin can abrogate unusual STAT3 activation via direct interaction and interfere with STAT3-mediated carcinogenesis [142]. Other research also showed that curcumin could inhibit PTC cell viability in a dose-dependent manner by stimulating caspase-mediated apoptosis and inhibiting constitutively active STAT3 by dephosphorylating Tyrosine 705 of STAT3 without altering STAT3 [145]. Our study illustrated that curcumin may strongly modulate STAT3, which had a binding affinity energy of −7.4 kcal/mol. Based on the PPI network analysis, STAT3 was recognized as a key target that could be harnessed for therapeutic purposes against liver cirrhosis.

## 5. Conclusions

This study looked at the possible effect of curcumin on the genes, proteins, and molecular pathways involved in the progression of liver cirrhosis and showed that curcumin may dramatically affect critical biological pathways associated with proteoglycans, AGE-RAGE, focal adhesion, and the HIF-1 signaling pathways in liver cirrhosis. Curcumin was shown to be likely to affect nine crucial genes/proteins concerned in this disorder, mostly expressed in the liver. Curcumin is proposed to have therapeutic benefits in different liver diseases from the literature, but more comprehensive molecular studies with robust patient cohorts in clinical trials are needed to confirm this efficacy.

## Figures and Tables

**Table 1:** The network analysis information of critical hub genes related to liver cirrhosis.

Ensembl Gene ID	Gene Symbol	Gene Full Name	Network Analyzer				
			Protein Class	Liver Specificity	Degree	Betweenness	Closeness
ENSG00000075624	ACTB	Actin beta	Plasma proteins	4.98	121	0.0332	0.4504
ENSG00000142208	AKT1	serine/threonine kinase 1	Enzymes	4.88	156	0.0403	0.4619
ENSG00000163631	ALB	Albumin	Plasma proteins	5.00	88	0.0493	0.4336
ENSG00000026508	CD44	CD44 Molecule	Blood group antigen proteins	4.83	88	0.0197	0.4219
ENSG00000168036	CTNNB1	Catenin beta 1	Plasma proteins	4.77	113	0.0276	0.4401
ENSG00000146648	EGFR	Epidermal growth factor receptor	RAS pathway related proteins	4.75	149	0.0311	0.4498
ENSG00000100393	EP300	E1A binding protein p300	Metabolic proteins	4.65	88	0.0172	0.4166
ENSG00000115414	FN1	Fibronectin 1	Plasma proteins	4.97	127	0.0408	0.4293
ENSG00000080824	HSP90AA1	Heat shock protein 90 alpha family class A member 1	Enzymes	4.98	95	0.0166	0.4285
ENSG00000254647	INS	Insulin	RAS pathway related proteins	4.00	92	0.0219	0.4295
ENSG00000177606	JUN	Jun proto-oncogene	Transcription factors	3.88	111	0.0224	0.4395
ENSG00000067560	RHOA	Ras homolog family member A	Enzymes	4.40	103	0.0239	0.4256
ENSG00000197122	SRC	non-receptor tyrosine kinase	Enzymes	4.64	168	0.0350	0.4612
ENSG00000168610	STAT3	Signal transducer and activator of transcription 3	Transcription factors	4.84	162	0.0279	0.4491
ENSG00000232810	TNF	Tumor necrosis factor	Plasma proteins	3.82	140	0.0314	0.4449
ENSG00000141510	TP53	Tumor protein p53	Transcription factors	3.72	115	0.0658	0.4412

## Data Availability

Not applicable.
